# Understanding litter decomposition in semiarid ecosystems: linking leaf traits, UV exposure and rainfall variability

**DOI:** 10.3389/fpls.2015.00140

**Published:** 2015-03-17

**Authors:** Aurora Gaxiola, Juan J. Armesto

**Affiliations:** ^1^Instituto de Ecología y BiodiversidadSantiago, Chile; ^2^Departamento de Ecología, Pontificia Universidad Católica de ChileSantiago, Chile

**Keywords:** C:N ratios, decomposition rates, ENSO, lignin, photodegradation, rainfall pulse, Chile

## Abstract

Differences in litter quality, microbial activity or abiotic conditions cannot fully account for the variability in decomposition rates observed in semiarid ecosystems. Here we tested the role of variation in litter quality, water supply, and UV radiation as drivers of litter decomposition in arid lands. And show that carry-over effects of litter photodegradation during dry periods can regulate decomposition during subsequent wet periods. We present data from a two-phase experiment, where we first exposed litter from a drought-deciduous and an evergreen shrub to natural UV levels during five, rainless summer months and, subsequently, in the laboratory, we assessed the carry-over effects of photodegradation on biomass loss under different irrigation treatments representing the observed range of local rainfall variation among years (15–240 mm). Photodegradation of litter in the field produced average carbon losses of 12%, but deciduous *Proustia pungens* lost >25%, while evergreen *Porlieria chilensis* less than 5%. Natural exposure to UV significantly reduced carbon-to-nitrogen and lignin:N ratios in *Proustia* litter but not in *Porlieria*. During the subsequent wet phase, remaining litter biomass was lower in *Proustia* than in *Porlieria*. Indeed UV exposure increased litter decomposition of *Proustia* under low and medium rainfall treatments, whereas no carry-over effects were detected under high rainfall treatment. Consequently, for deciduous *Proustia* carry-over effects of UV exposure were negligible under high irrigation. Litter decomposition of the evergreen *Porlieria* depended solely on levels of rainfall that promote microbial decomposers. Our two-phase experiment revealed that both the carry-over effects of photodegradation and litter quality, modulated by inter-annual variability in rainfall, can explain the marked differences in decomposition rates and the frequent decoupling between rainfall and litter decomposition observed in semiarid ecosystems.

## Introduction

Environmental controls on litter decomposition in terrestrial ecosystems are fundamentally related to differences in litter quality (e.g., element concentration in tissues) and local climate (temperature, precipitation). These two factors modulate microbial (and other decomposers) activity and thus mediate the processing of organic matter and the rates of internal ecosystem nutrient cycling (Meentemeyer, 1978; Aber et al., 1990; Couteaux et al., 1995; Aerts, 1997; Gholz et al., 2000; Hättenschwiler and Jørgensen, 2010). Nutrient concentrations (e.g., percent nitrogen), carbon-to-nitrogen (C:N) ratios, or lignin content of senescent foliage are all good indicators of litter quality for decomposers (Melillo et al., 1982; Parton et al., 2007). Local climatic variables such as temperature and moisture availability (Davidson and Janssens, 2006) regulate soil microbial activity through their effects on enzyme kinetics and nutrient diffusion. In semiarid ecosystems the interplay among all these and other variables affecting decomposition remains little understood. Understanding the drivers and mechanisms of nutrient cycling represents a conspicuous gap in our knowledge of the dynamics and functioning of semiarid ecosystems. Recent studies have suggested that factors, such as litter layer thickness (Henry et al., 2008), litter spatial distribution (Austin et al., 2009), soil microbial films (Barnes et al., 2012), and exposure to natural levels of UV radiation (Austin and Vivanco, 2006; Brandt et al., 2007, 2009) could also modulate litter decomposition rates and nutrient cycling in deserts.

Litter decomposition rates in semiarid ecosystems can be extremely variable (Zhang et al., 2008) and not directly dependent on precipitation, as for the same amount of rainfall (i.e., 250 mm) litter decay rates (i.e., *k*) can vary from 0.1 to 1.2 y^-1^ (Austin, 2011). Furthermore, decomposition studies have failed to document correlations between litter mass loss and differences in litter quality among species (Schaefer et al., 1985; Martínez-Yrízar et al., 2007; Austin, 2011). Considering that neither litter quality nor precipitation can fully account for the large differences in litter decay rates observed among semiarid ecosystems (Vossbrinck et al., 1979; Whitford et al., 1986; Austin and Vivanco, 2006; Gallo et al., 2006; Brandt et al., 2007; Zhang et al., 2008) other factors must be involved. This paper will explore how an additional factor, photodegradation, could play a role, in combination with litter quality and precipitation, to explain the observed variability in decay rates. Here we will focus primarily on the role of natural UV radiation during rainless periods, which is known to enhance the rates of biomass loss via photodecomposition or photodegradation, through the physicochemical breakdown of organic carbon compounds as a result of absorption of UV by litter (Vossbrinck et al., 1979).

High levels of solar radiation have for long been associated with carbon losses from organic matter especially in semiarid ecosystems (Pauli, 1964; Moorhead and Callaghan, 1994). However, until recently, studies have started to quantify the role of UV radiation on litter biomass losses. A field study in the Sonoran desert, estimated that 18–22% of litter mass loss of the evergreen shrub *Larrea tridentata* was attributable to near-ambient solar UV radiation (Day et al., 2007). These authors identified lignin as the main organic compound lost from litter under natural UV action. Another study in California grasslands showed that grass and forb litter lost from 8 to 10% of their initial mass when exposed to natural solar radiation. Moreover, grass litter experienced >50% reductions in lignin content (Henry et al., 2008). These studies indicate that natural UV exposure can modify litter quality by changing the concentration of aromatic and recalcitrant compounds, such as lignin, which are hard to decompose by soil microorganisms (Melillo et al., 1982; Gallo et al., 2009; Foereid et al., 2010). Recent studies have therefore started to suggest that the magnitude of UV-exposure effects on litter mass loss seems to be modulated by differences in leaf litter chemistry. For instance, a field experiment along an aridity gradient in North America (Gallo et al., 2009) showed greater mass loss due to UV exposure from high- than from low-quality litter (i.e., low lignin: N *vs.* high lignin: N ratios, respectively). These authors also found that mass loss from UV-exposed litter was more than twice as high as mass loss from UV-protected litter. However, the authors did not detect significant differences in decomposition rates between UV-exposed and UV-protected litter at sites receiving higher rainfall. Accordingly, exposure to UV radiation during dry periods can have significant positive effects on decomposition when microbial activity is limited by aridity (Henry et al., 2008). In a lab experiment, pre-exposure to artificial UV enhanced the digestibility of a C4 grass when subsequently decomposed under wetter conditions that favored microbial activity (Foereid et al., 2010). The authors suggested that exposure to UV radiation during dry periods was like a pre-treatment for decomposition during wetter conditions. Hence, carry-over effects of exposure to natural UV radiation could be more important in seasonally dry environments.

Enhanced decomposability after dry periods, however, can additionally depend on initial differences in leaf litter chemistry, which could interact with the subsequent microbial activity (Gallo et al., 2009; Foereid et al., 2010). We suggest therefore that carry-over effects of UV radiation on overall litter decomposition may depend not only on rainfall distribution and length of the dry period, but also on differences in leaf traits among coexisting species. This paper presents an experiment aimed at disentangling the relative contributions of photodecomposition, litter quality, and water supply (due to pronounced differences in rainfall among seasons or years) on litter decomposition in semiarid ecosystems. Additionally, we discuss how the interactions among these drivers could account for the high variability in decomposition rates reported in the literature for semiarid ecosystems.

The study was conducted at the southern margin of the Atacama Desert, where rains are restricted to the austral winter months (June–August) and the annual amount of rainfall varies greatly (<10–300 mm per year) in response to the climate cycles associated with El Niño Southern Oscillation (ENSO; Jaksic, 2001; Jiménez et al., 2011). We addressed the following hypotheses about litter decomposition in this semiarid environment: (i) Photodecomposition effects during the dry period will carry-over onto subsequent wet period, but such effects may be a function of litter quality; (ii) for a given litter quality the relative importance of carry-over effects would be higher in drier than in wetter years.

## Materials and Methods

### Study Site and Plant Species

Our study was conducted in the scrublands of Fray Jorge Forest National Park (30° S, 71° 40^′^ W), Chile. The local climate is Mediterranean semiarid (López-Cortés and López, 2004), with extremely dry periods extending for 6–7 months (November–April) without rain, followed by humid winters (May–August) when >95% of the rain falls. Average annual rainfall for the last 25 years in the study area is 151 ± 43 mm (mean ± 1 SE, CV = 74%, Gaxiola et al., unpublished data). Higher annual rainfall is largely associated with extreme positive phases of ENSO. For example, 1991–1992 and 1997 have been identified as El Niño years with annual rainfall between 240 and 300 mm. In contrast, years 1988 and 1990 were extremely dry, with an annual rainfall of 11 and 33 mm respectively, and associated with the negative phase of ENSO, or La Niña years (Gutiérrez et al., 2010; Jiménez et al., 2011). Regarding the focal driver of our study, UV radiation can be extremely high in this area of northern Chile, particularly during the cloudless summer months. According to the Direccion Meteorológica de Chile (DMC; http://www.meteochile.gob.cl/radiacion_uv.php) the UV index for our study site can be higher than 11 at midday during summer months. Similarly, we measured UV radiation in our study site with broadband UV sensors calibrated for 280–320 nm (SU-100, Apogee Instruments Inc., Logan, UT, USA) and recorded unweighted UV mean values of 55 Wm^-2^ during January with an absolute maximum of 62.4 Wm^-2^ (unpublished data).

Predominant vegetation of the study area is a mixed scrubland characterized by the dominance of evergreen and summer-deciduous shrubs, 1–3 m in height, with a seasonal ground layer of annual species. The thorny evergreen shrub *Porlieria chilensis* IM Johnst. (Zygophyllaceae) dominates the overstory layer with 25–35% cover, along with two deciduous species, *Proustia pungens* D. Don (Asteraceae; 10–20% cover), and *Adesmia bedwellii* Skottsb (Leguminosae; 3–6% cover), leaving ample open spaces between shrubs (Gutiérrez et al., 1993). For the analysis of litter decomposition, we selected two of the dominant shrub species, with contrasting leaf habits: (1) the evergreen *Porlieria chilensis* and (2) the drought-deciduous *Proustia pungens* which are henceforth referred to by genus only. These two shrub species differed in leaf mass per area (LMA), foliar nitrogen and carbon content (leaf-N and leaf-C, respectively), lignin content, and nutrient resorption proficiency (Table S1). We compared litter degradation in these two species not only because these dominate the semiarid vegetation, but also because differences in green leaf traits can persist in senescent leaves and may in turn promote differences in litter quality and decay rates in the field (Cornwell et al., 2008). Leaf litter tends to accumulate under the crown foliage, which is absent during summer in the case of *Proustia*. Hence leaves of *Proustia* are exposed to full sun for during the dry season.

### Collection of Plant Material

We collected green leaves and litter from 20 randomly selected adults of each shrub species within an area of about 3 hectares. At least 10 g of green mature foliage and at least 20 g (dry weight) of recently fallen dead leaves were collected from under each individual. Green leaves were collected by hand in mid September. To facilitate collection of recently abscised leaves, we placed small litter collectors under each shrub, cleaned weekly to minimize sun exposure of fresh litter. Litter collectors were placed in late spring (mid October) and removed before the onset of summer (early December).

At least 20 leaves per individual were scanned with a HP DeskScan II scanner (Hewlett-Packard, Palo Alto, CA, USA), and images saved at 1200 dpi. Mean leaf area was estimated by counting black pixels on black and white scanned images using Adobe Photoshop 90 (Adobe Systems Incorporated_,_ USA). Leaves were weighed after drying at 65° C for 2 days (or until constant weight). Data on leaf mass and area for each species were used to calculate LMA. A total of 14 samples of dried green and dead leaves per species were ground to determine total C and N concentrations by flash combustion using a NA2500 Carlo Erba Element Analyzer. Lignin content was determined following Van Soest (1963), using one gram of ground litter milled to 1-mm mesh size for acid detergent fiber (ADF). Litter-N concentrations were interpreted as resorption proficiency (*sensu* Killingbeck, 1996) after we corrected for changes in LMA, to control for senescence-related differences in leaf carbon.

### Photodecomposition Field Experiment

To assess the effects of natural UV exposure on litter biomass loss for the two shrub species, we established a photodecomposition experiment in the field. The experiment lasted 5 months (December through April), including the entire rainless summer period in semiarid Chile. During the 5 months, we were able to assess the direct effects of natural UV exposure on leaf litter dynamics in the complete absence of rainfall. For this experiment, litter samples (about 5 g) were introduced in 15 × 30 cm litterbags built with a plastic sheet (UV filter or UV-transmitting film) on the upper side and a plastic fiberglass mesh (2 mm) in the back; mesh at the bottom provided ventilation and avoided overheating of the litterbag contents. We manipulated UV exposure of the litterbags by using UV-blocking or UV-passing plastic sheets on the sun-exposed side. Solar UV radiation was filtered out by using 125 μm UV-blocking film (Mylar-type Cadco clear polyester film, Cadillac Plastic and Chemical, Phoenix, AZ, USA; sharp transmission cutoff below 325 nm). For the second group of litterbags, we used Plexiglas UV-transmitting film with a sharp transmission cutoff below 275, thus allowing for >95% transmission of the UV-waveband. Litterbags with UV-passing plastic represented “control” conditions (UV+), and litterbags with Mylar plastic filter represented reduced UV radiation (UV-) treatment in the field.

We placed all 180 litterbags, constructed as indicated above (two UV-treatments, two species, 45 replicates), on randomly chosen areas located between shrubs (no shrub cover directly above), about 3–4 m away from shrubs from which leaf samples were originally collected. Bags were placed in the field in December (onset of the dry period) and kept within the boundary of the 3-hectares study area. We removed dead annual plant material that could cast shade on litterbags. To avoid contact between litterbags and the soil directly underneath we lifted each litterbag with wires and anchored them at ∼20 cm above the soil level. Photodecomposition thus reflects only the loss of mass due to solar radiation received by the bags. All litterbags were retrieved in the midst of the austral autumn (late April), 5 months after the initiation of the experiment, and before the onset of the rains. Eight damaged or broken litterbags were eliminated from the experiment. Contents of each litterbag were placed inside sealed plastic bags to avoid dead matter loss and taken to the laboratory for dry mass estimates. These contents were oven-dried at 60° C for 2 days and subsequently removed from the mesh and weighed. We took subsamples from all litterbags for determination of total C and N. Subsamples of material from 26 litterbags (per treatment and species) were used for lignin determinations. Using these values we calculated litter-C, litter-N, litter C:N, and lignin:N ratios for UV+ and UV- treatments and for each of the two shrub species. All chemical analyses were conducted in the laboratory of Agro-analyses from the Faculty of Agronomy and Forest Research, Catholic University of Chile. Photodecomposition effect was estimated as the percentage of litter mass loss over the 5 months period of exposure to full sun. Biomass loss % = ((initial weight – final weight) /initial weight)^∗^100.

Every month during the field experiment we monitored temperatures inside 10–15 litterbags per treatment, five times in 1 day, by sticking a mini *k*-thermocouple probe inside the bags. The thermocouple was connected to a SM320 datalogger (Dickson Addison, USA).

### Laboratory decomposition experiment

To test the predictions about the combined effects of photodecomposition and soil moisture on litter decay, we conducted a follow-up decomposition experiment in the laboratory. For this experiment we used litter subsamples taken from the UV- or UV+ litterbags from the photodecomposition experiment. We weighted these subsamples in order to get at least 3 g of initial dry weight. Each litter sample was placed over a 5 × 5 cm square of 2-mm green-fiberglass mesh on the surface of small plastic trays (15 × 15 cm and 5 cm-depth) containing 350 g of soil collected from the study site. Each litterbag was slightly covered with a less than a teaspoon of soil, as soil particles may promote litter biomass loss (Moorhead and Reynolds, 1991; Barnes et al., 2012). In August–September the total of 132 leaf litter mesh bags (2 species × 2 UV treatments × 3 simulated rainfall levels, 11 replicates) were randomly distributed over benches in a controlled temperature-room, where temperature was set at 23° C. Litter samples were assigned one of the three water treatments that simulated actual ranges of annual rainfall recorded over the past 25 years in the study area: (1) low rainfall or “dry year” (water addition equivalent to 15 mm of rain), (2) average or “normal year” (equivalent to 140 mm), and (3) high rainfall or “wet year” (equivalent to c. 250 mm). The latter amount resembles the rainfall of an El Niño event. According to the 25-years instrumental rainfall records for the study site, in dry years an average rainfall event is about 0.2 ± 0.1 mm per day (or ∼0.02 ml cm^-2^); for an average year, average daily pulses are 1.1 ± 0.3 mm (or ∼0.11 ml cm^-2^), and can last 3–6 h; and finally, average pulses on wet years are 2.5 ± 0.4 mm (or ∼0.25 ml cm^-2^) spread over 10–15 h (Gaxiola et al., unpublished data). Based on this information we established the amount and frequency of water pulses for each water treatment. Water treatments were provided in ml cm^-2^, as 0.1 ml cm^-2^ is equivalent to 1 mm of rainfall. For the low rainfall treatment, and considering the size of the plastic trays (i.e., 15 × 15 cm) we added pulses of 0.02 ml cm^-2^. The equivalent of 15 mm of rain was added after 75 pulses over 2 months. For this treatment we used a pipette. For the treatment equivalent to an average year, pulses of water ranged from 0.5 to 1.1 ml cm^-2^ until the equivalent of 140 mm of rainfall was accumulated (after 19 pulses of different magnitudes). To simulate the condition of a wet El Niño year, we added pulses ranging from 0.8 to 2.5 ml cm^-2^ until the equivalent of 250 mm was accumulated (i.e., 18 different pulses). For the last two treatments we used trigger water sprayers of different sizes to control the water volume reaching the bags. Water pulses were randomly distributed over the first 4 months of the experiment, with 80% of the pulses concentrated in the second and third months, as climatic measurements in our study site show that rains are greatest in June and July (80% of rainfall).

Every 20 days we shuﬄed litterbags to reduce effects of position along benches. Six months later, litter samples were oven-dried at 60° C until constant mass. Biomass loss was monitored over 6 months since the experiment was initiated in the laboratory. Litter decomposition was estimated as the percentage of remaining biomass. Remaining biomass % = (final weight/initial weight)^∗^100.

### Data Analyses

To assess inter-specific differences in foliar traits for green leaves and leaf litter before and after the photodecomposition experiment, we used pairwise comparisons based on Welch two sample *t*-tests. Effects of differential exposure to natural UV radiation on litter biomass loss were tested using a two-way analysis of variance (ANOVA; Crawley, 2002), with species and UV-treatments as factors.

The laboratory experiment on litter decomposition under different levels of soil moisture was analyzed with a generalized linear model (*glm* routine in *R*) to test for the effects of the three irrigation treatments, the carry-over effects of previous photodecomposition in the field (UV treatments), species identity, and litter quality (i.e., litter C:N and lignin:N ratios). We also tested for the interactions among these factors. Decomposition, expressed as remaining litter biomass (%) was the response variable in the analysis. A full model was fitted to the data and then reduced to a minimal adequate model by removing terms sequentially, starting from the highest-order interaction terms, and using *anova* command in R to test for statistical significance of each model term (Crawley, 2002). All data were checked for homogeneity of variances before analyses and appropriate transformations were used as needed. Analyses were done with *R* (R Development Core Team, 2011).

## Results

### Traits of Green Leaves and Litter

*Proustia* and *Porlieria* differed in green leaf traits in accordance with their contrasting leaf habits. LMA, lignin% and leaf carbon content as well as C:N and lignin:N ratios were lower for the deciduous *Proustia* than for the evergreen *Porlieria*, but total N content was higher in *Proustia* leaves (Table S1). With regard to litter chemistry, C and N contents, and C:N ratios did not differ between species, and only lignin and lignin:N ratios were higher for *Porlieria* than for *Proustia* litter (Table S1). Lower litter-N content in *Proustia* was associated with a strong N-resorption proficiency (i.e., 46%), which was higher than that of *Porlieria* (25% N resorption; *t*= 5.9, df = 32, *P <*0.0001). Reductions of litter-N in *Proustia* resulted in similar C:N ratios in the litter of both species (Table S1). Lignin:N was higher for *Porlieria* than for *Proustia* and this difference together with contrasts in LMA could represent a major disparity in litter quality between the two focal species.

### Photodecomposition in the Field

**FIGURE 1:**
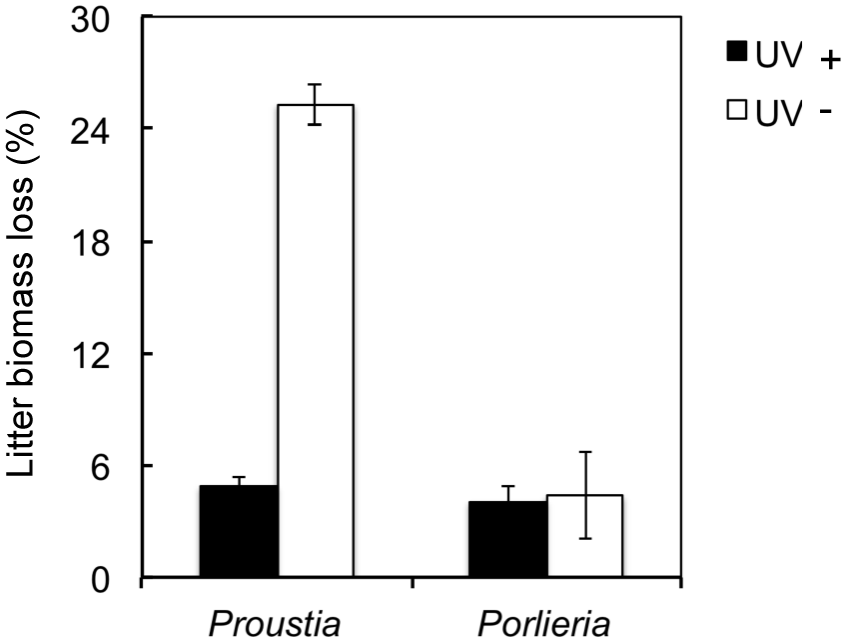
**Effects of UV exposure on litter biomass after a 5-months experiment in semiarid Chile.** Data shown for deciduous *Proustia* and evergreen *Porlieria*. UV- represents a reduction of 96–98% of natural UV levels; UV+ is the control treatment without UV filters. Data are means ± 1 SE (thin bars).

Natural UV radiation significantly reduced litter biomass as seen when data for both species were analyzed together (UV+ = 14.8 ± 1.3 *vs.* UV- = 4.7 ± 0.13; *F_1,136_* = 144.6, *P <*0.0001). We found higher biomass loss in *Proustia* than in *Porlieria* (*Proustia*= 15.1 ± 1.2 *vs. Porlieria* = 4.2 ± 0.21; *F_1,135_* = 5.6, *P <*0.001). In UV+, the litter mass loss for *Proustia* was almost 25% whereas for *Porlieria* it was less than 5% (**Figure 1**). The interaction term ‘species × UV treatment’ was significant (*F_1,132_* = 57.3, *P <*0.0001). Comparisons between initial and final litter mass weights for UV- treatment showed no significant differences either for *Proustia* (5.34 ± 0.02 g vs. 5.18 ± 0.02 g; *t* = 4.11, d.f. = 70, *P* = 0.16), or *Porlieria* (5.01 ± 0.15 g vs. 4.87 ± 0.01 g; *t* = 0.87, d.f. 70, *P* = 0.13). Therefore our UV-treatment indeed reduced UV radiation and protected litter from photodegradation.

Overall, photodecomposition changed litter carbon and lignin content altering litter chemistry, particularly in the deciduous species. *Proustia* litter had lower carbon and lignin content in UV+ than in UV- treatments (**Table 1**) and such reduced carbon content resulted in lower C:N ratios and lignin:N in the UV+ treatment (**Table 1**). In the case of *Porlieria,* we found no differences in litter chemistry between UV treatments (**Table 1**). However, we noted that the initial lignin content in *Porlieria* decreased from 12.46 to 11% in both treatments (Table S1 and **Table 1**, respectively), suggesting that exposure to natural solar radiation changed its litter chemistry. Finally, we point out that because there were no changes in litter N% between UV treatments for either species (**Table 1**), subsequent changes in litter chemistry could be attributed to carbon losses only.

**Table 1:** Comparisons of leaf litter traits between *Proustia* and *Porlieria* following 5 months of photodecomposition in the field.

	*Proustia*			*Porlieria*		
Trait	UV-	UV+	*t* test *(d.f.)*	UV-	UV+	*t* test (*d.f.*)
LMA (g. m^-2^)	143.2 (3.6)	129.7 (1.65)	6.44 (70)***	264.8 (3.59)	263.1 (4.07)	0.31 *(70)*
C%	49.4 (0.49)	38.1 (0.33)	18.5 (63)***	51.1 (0.57)	50.5 (0.25)	1.86 *(54)*
N%	1.07 (0.07)	1.06 (0.04)	0.03 *(64)*	1.11 (0.11)	1.12 (0.09)	1.00 *(70)*
C:N	45.5 (0.57)	35.3 (0.32)	14.9 (57)***	46.8 (0.68)	44.3 (0.39)	1.99 *(58)*
Lignin (%)	9.87 (0.13)	7.36 (0.14)	14.2 (72)***	11.17 (0.19)	11.05 (0.15)	0.55 *(72)*
Lignin:N	9.34 (0.12)	6.82 (0.13)	13.4 (72)***	10.05 (0.18)	9.80 (0.16)	1.01 *(70)*

Intraspecific comparisons of litter traits after a rainless period of exposure to natural UV radiation in semiarid Chile (UV- implies a reduction of 96–98% of natural UV radiation; UV+ is the control treatment with less than 10% reduction of UV levels). Values are means ± 1 SE, and student-*t* tests are given for intraspecific comparisons. ***P < 0.0001.

During the 5-months photodecomposition experiment, we detected minor increases in temperature inside the litterbags (*F_1,4_* = 2.5, *P* = 0.044; Figure S1) and these were associated with higher maximum temperatures in the summer. Thermal environments did not differ between UV treatments (*F_1,1_* = 0.11, *P* = 0.25; Figure S1) and we must rule out temperature effects on carbon losses.

### Laboratory Decomposition Experiment

**FIGURE 2:**
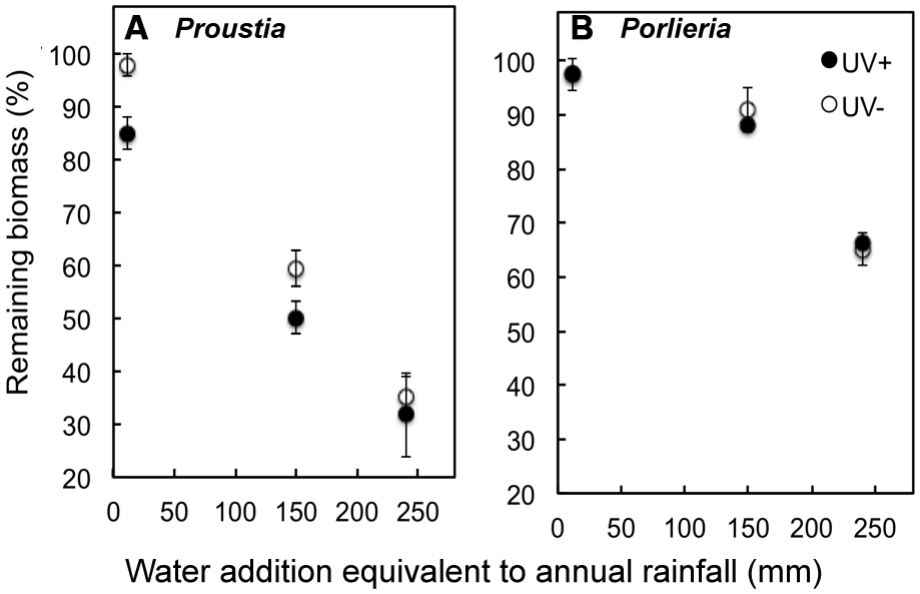
**Remaining litter biomass (%) at the end of a 6-month decomposition experiment, preceded by photodegradation in the field.** Samples from the field experiment are shown by open (UV+) or filled symbols (UV-). The laboratory experiment included irrigation treatments representing years with contrasting annual rainfall. Irrigation treatments represented the equivalent rainfall of a dry year (˜15 mm), an average year (140 mm) and wet (El Niño) year (240 mm) in semiarid Chile. The experiment was conducted under constant temperature (23°C). **(A)** Remaining litter biomass *Proustia* and **(B)** *Porlieria*. Data are means ± 1 SE.

Between 2 and 65% of initial litter mass was lost during the subsequent 6-months of decomposition in the laboratory after water addition. Variation in biomass loss among litter samples was associated primarily with differences among irrigation treatments (i.e., simulated rainfall), but we also detected the carry-over effects from the process of litter photodegradation in the previous field experiment, and effects of species identity (**Figure 2**).

Experimental water supply strongly enhanced the decomposition of litter samples of both species (*F_1,130_*= 283, *P* < 00001) and water supply alone explained 57% of total model deviance. When all data were pooled there were no differences in remaining biomass between UV treatments across all water addition treatments (UV+: 73.1% ± 2.9 and UV-: 77.3% ± 3.2; *F_1,129_*= 1.45; *P* = 0.22). This could be a consequence of the marked differences in remaining biomass between shrub species (*F_1,128_*= 113, *P* < 0.0001; **Figure 2**). The largest difference in litter decomposition between the two shrub species was due to the “high rainfall” treatment (i.e., water addition equivalent to 250 mm). In this treatment, at the end of the lab experiment, it remained 75% of the initial mass of *Porlieria* and only 25% of the mass of *Proustia*. This difference was statistically accounted for the significant interaction term ‘species × water treatment’ (*F_1,126_* = 103, *P* < 0.0001).

**FIGURE 3:**
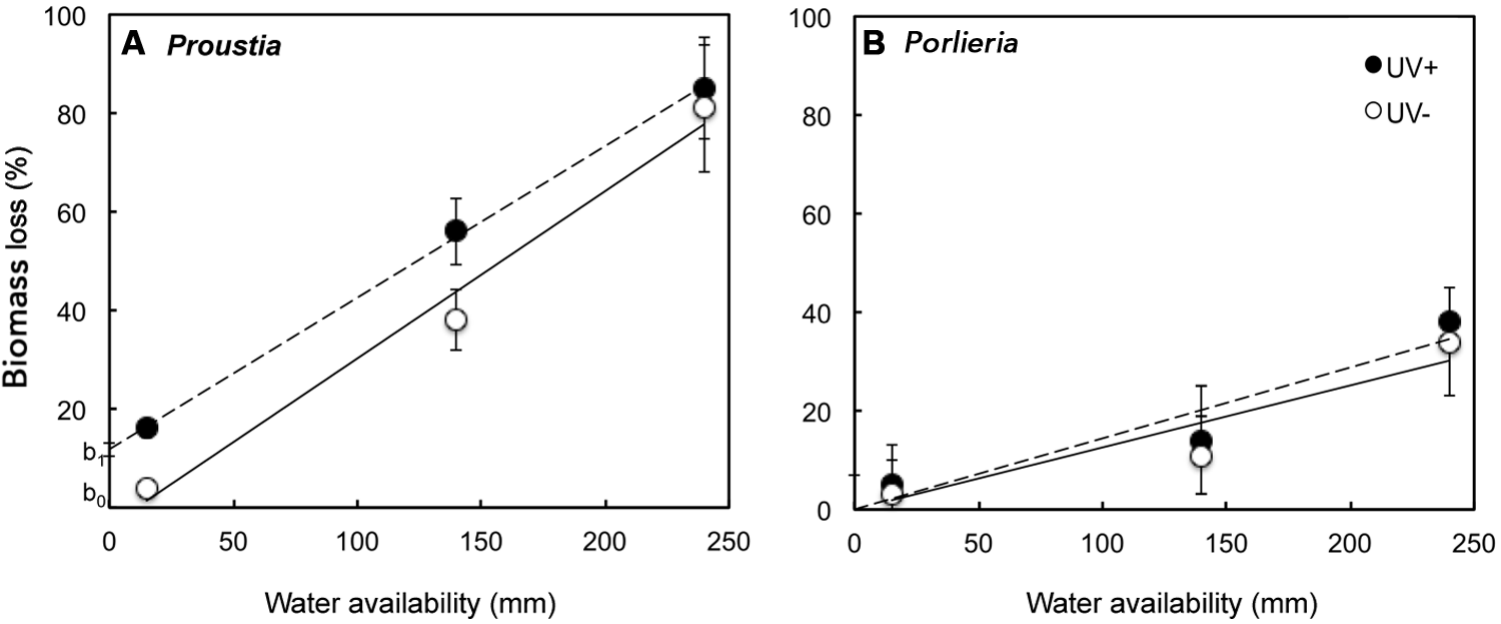
**Graphic representation of carry-over effects of photodecomposition on litter biomass losses (%) as a function of water availability for two shrub species with contrasting leaf traits. (A)** Carry-over effects of UV exposure during the dry summer months promoted further carbon losses from *Proustia* litter, particularly in the irrigation treatments equivalent to low and average rainfall years, **(B)** No significant effects of photodecomposition on further biomass losses are observed in the case of *Porlieria* litter. Lines represent significant relationships between experimental irrigation treatments and biomass loss; dashed lines = UV+ (*Proustia*: *y* = 0.32 ± 0.006x + 12.9 ± 1.07; *R^2^* = 0.95 and *Porlieria*: *y* = 0.123 ± 0.01x; *R^2^*= 0.91) and solid lines = UV- (*Proustia*: *y* = 0.29 ± 0.01x; *R^2^* = 0.95 and *Porlieria*: *y* = 0.122 ± 0.01x; *R^2^* = 0.90).

Pre-exposure to UV radiation in the field enhanced litter decomposition only for *Proustia* (**Figure 2A**). This effect accounted for the significant interaction term ‘species × UV treatment’ (*F_1,125_* = 55, *P* < 0.01). Remaining biomass was lower in *Proustia* litter in the low and medium irrigation treatments when previously exposed to UV, but no differences in decomposition between UV+ and UV- were observed for the “high rainfall” treatment (**Figure 3A**). The triple interaction term ‘species × UV treatment × water treatment’ was not significant (*F_1,124_* = 0.38, *P* = 0.5). Hence, as expected, carry-over effects of photodegradation in *Proustia* decreased with increasing water supply. *Porlieria* in turn showed no differences either in the slope or the intercept of the carbon loss vs. water availability relationships between UV treatments (**Figure 3B**).

## Discussion

Our study offers experimental evidence that interspecific differences in litter quality, in combination with natural UV exposure during dry periods and mass loss during wet periods can promote broad variance in decomposition rates in semiarid ecosystems (**Figure 3**). Broad variance in decomposition rates has been reported in the literature for semiarid ecosystems around the globe (Zhang et al., 2008).

We show here that litter from drought-deciduous *Proustia* decayed more rapidly than litter from evergreen *Porlieria*, which had higher LMA, lignin% and lignin:N ratios than *Proustia* (**Table 1**). Differences in litter chemistry accounted for the marked contrast in decomposition rates between species and among irrigation treatments, independently of pre-exposure to UV radiation (**Figure 3**). Furthermore, we were able to show that these two coexisting shrub species were able to take advantage of contrasting abiotic factors that promote decomposition in arid lands. In the case of *Proustia,* UV exposure changes litter quality, which during the wet season can degrade even at low levels of annual rainfall or during dry years (**Figures 2** and **3**). Therefore, for *Proustia,* litter pre-exposure to UV radiation produce carry-over effects that promote ensuing microbial decomposition. On the other hand, litter from *Porlieria* which tends to accumulate directly below the shade of the shrub crown, relies primarily on microbial decomposition and thus on soil moisture levels that promotes microbial activity.

Foliar traits and litter chemistry strongly influence the breakdown of litter by UV exposure (Gallo et al., 2006; Brandt et al., 2007; King et al., 2012); indeed, litter exposure to 5 months of natural UV radiation had strong photochemical effects on *Proustia* but negligible effects on *Porlieria* (**Table 1**). A high proportion of lignin and carbon was lost from *Proustia* litter during UV exposure in the field, whereas litter chemistry of evergreen *Porlieria* did not differ between UV treatments (**Table 1**). Differences in litter biomass loss between the two focal shrub species, could also be accounted for by other factors such as leaf size, which has been shown to influence UV exposure effects on litter decomposition (Gallo et al., 2006; King et al., 2012; Song et al., 2013). *Proustia* leaves (2 cm^2^) are much larger than those of *Porlieria* (<0.5 cm^2^) and hence on the basis of differences in leaf area we could expect higher effects of ambient UV on *Proustia* litter. Leaves of *Porlieria* remain protected under evergreen foliage after falling, but are exposed to high solar radiation levels for long periods while still on the plant. It has been reported that long exposure of green foliage to UV can induce the production of photo-protective compounds, including flavonoids and polyphenols such as tannins (Rozema et al., 1997; Gehrke, 1999). These photo-protective compounds can remain active long after leaf abscission and thus reduce the effect of UV exposure on the degradation of lignin and other carbon compounds.

Our data show that litter of *Porlieria* had small but non-significant carbon losses during the field experiment (cf. values in Table S1 *vs*. those in **Table 1**). This result suggests that sunlight caused only weak degradation of *Porlieria* litter. Because lignin is a light absorbing molecule (Austin and Ballaré, 2010), it is the most likely carbon compound degraded by sunlight during our field experiment, and according to these authors solar wavelengths such as blue–green quanta can also promote carbon losses in leaf litter. On these grounds, it is likely that in addition to UV, other solar wavelengths may promote the breakdown of carbon in *Porliera* litter. We cannot document these effects because the radiation filters that we used in the photodecomposition field experiment only blocked UV-B radiation. Furthermore, cellulose and hemicellulose are also susceptible to breakdown by solar exposure (Brandt et al., 2010; King et al., 2012) and photodegradation of these compounds could also contribute to carbon losses observed in *Porlieria* and in *Proustia* litter. Further chemical analyses for cellulose and hemicellulose are required to evaluate the extent to which the interaction between solar wavelengths and carbon compounds may account for the differential effects of solar radiation on litter chemistry of coexisting shrub species.

In the lab experiment, which represented the humid phase of the decomposition process in semiarid ecosystems, litter from *Proustia* decayed more rapidly than litter from *Porlieria*. To explain this result, we argue that contrasting litter chemistry promoted differences in litter decomposition between species across “rainfall” treatments (**Figure 2**). These results agree with studies highlighting the importance of litter chemistry on litter decomposition due to positive effects of low C:N and lignin:N ratios (Murphy et al., 1998; Pérez-Harguindeguy et al., 2000). This could be associated with the fact that litter C:N of *Proustia* was close to 30, and according to a recent review it is close to the optimal C:N ratio for the microbial decomposers (Manzoni et al., 2010). Results from the lab decomposition experiment are in conflict with studies that did not find a strong or significant correlation between litter biomass loss and precipitation amount (Martínez-Yrízar et al., 2007). In the present study, biomass losses were strongly and positively correlated with increasing simulated “rainfall” (**Figure 2**). However, we found that for the same water treatment there were marked differences in biomass loss between *Proustia* and *Porlieria* (**Figures 2** and **3**). It is worth noting that litter biomass losses for *Proustia* followed the classical negative exponential function reported for mesic ecosystems, whereas biomass losses from *Porlieria* litter followed the linear function described for semiarid ecosystems (Austin and Vivanco, 2006; Barnes et al., 2012).

Carry-over effects of photodegradation were only noticeable under low irrigation and these effects became negligible when water supply simulated a high rainfall year (**Figure 3A**). This result is consistent with our predictions about the relative importance of carry-over photodegradation effects on litter decomposition. In this context, we show that during the dry season litter of *Proustia* was pre-degraded by natural exposure to UV (and possibly other solar wavelengths) to make it less rich in carbon and recalcitrant compounds (**Table 1**). Such chemical changes made litter of *Proustia* more readily degradable by microorganisms under the treatments that simulated low or average rainfall; in the laboratory; in other words, carry-over effects of photodegradation changed the intercept at which litter decomposition started under humid conditions (from b0 to b1 see **Figure 3A**). Therefore, for a given amount of rainfall, *Proustia* litter decomposed faster when previously exposed to UV during the dry summer (**Figure 2A**). Our results provide indirect evidence that litter photodecomposition during extended dry periods could enhance microbial activity during subsequent rainy seasons in “normal” or dry years (**Figure 3A**). We also show that during high-rainfall years (i.e., El Niño years) when this semiarid ecosystem receives over 250 mm, carry-over effects of previous photodegradation become irrelevant (**Figure 2A**). In recent decades, annual rainfall has declined significantly in this semiarid region (Jiménez et al., 2011), and season as low rainfall years increase in frequency, carbon losses due to photodecomposition during the prolonged dry will become an increasingly important driver of overall litter decomposition and ecosystem nutrient recycling.

These findings are consistent with a model of litter decomposition in arid environments where different leaf traits have strong effects on soil organic matter turnover (Cornwell et al., 2008) and where shrub-induced spatial heterogeneity of soil nitrogen and carbon availability in the ecosystem can strongly affect microbial processes (Gutiérrez et al., 1993; Aguiar and Sala, 1999). In arid regions that are affected by variable climate systems, such as ENSO, photodecomposition will vary greatly in its importance on overall litter degradation depending on inter-annual fluctuations in rainfall. In dry years, or during long dry spells, litter chemistry will be largely dependent on slow photodecomposition processes modulated by UV exposure on the ground. This is a driver through which litter of some species (e.g., drought-deciduous *Proustia* in this study) could degrade significantly even at low levels of annual rainfall over several dry years (**Figures 2** and **3**). This mechanism could facilitate the access of deciduous shrub species to recycled nutrients even during low rainfall (La Niña) years. In contrast, evergreen species such as *Porlieria* are strongly dependent on high-rainfall (El Niño) years for complete turnover of their organic matter, as facilitated by enhanced microbial activity. We conclude therefore that coexisting shrub species with contrasting leaf traits in semiarid ecosystems can take advantage of different abiotic settings to promote local nutrient cycling. We propose that such interspecific differences, added to temporal and spatial variability in resources and water supply, could enhance the variability in decomposition rates that has been reported for semiarid ecosystems, as well as cause the decoupling between annual rainfall and the rates of litter biomass loss.

## Author Contributions

AG designed and performed all experiments and co-wrote the paper with JA.

## Conflict of Interest Statement

The authors declare that the research was conducted in the absence of any commercial or financial relationships that could be construed as a potential conflict of interest.
