# Supplementary material for: Understanding litter decomposition in semiarid ecosystems: linking leaf traits, UV exposure and rainfall variability
Source: Front Plant Sci. 2015 Mar 17;6:140. doi: 10.3389/fpls.2015.00140 (PMC4362295; doi:10.3389/fpls.2015.00140)
Supplement: Supplementary file 1 [file presentation_1.pdf]

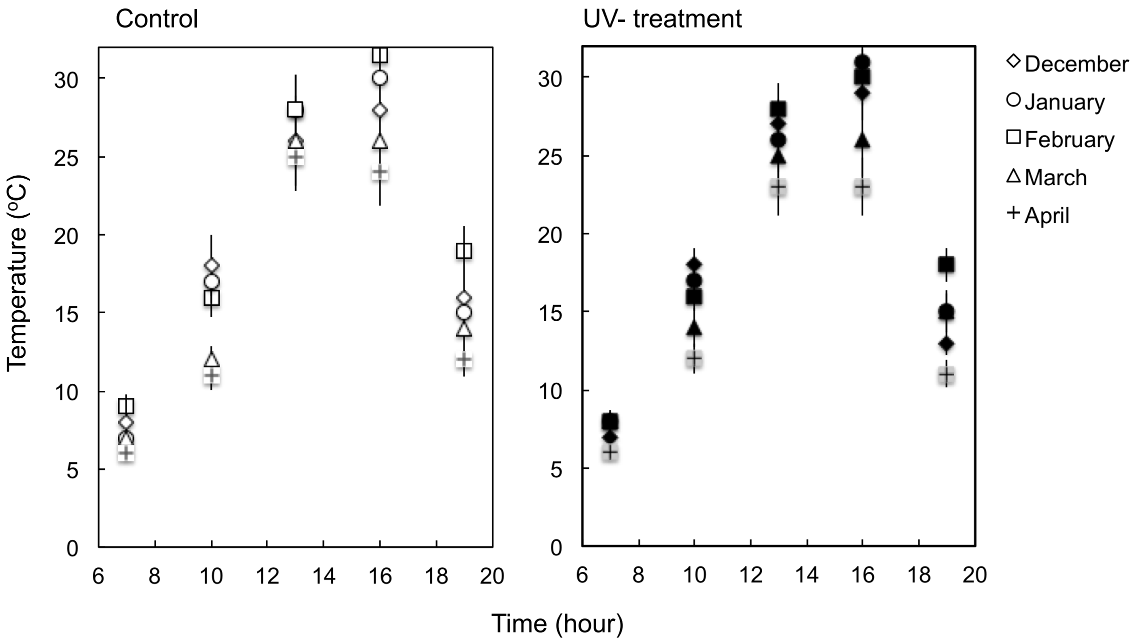

Figure S1 Mean temperatures (± 1 SEM) inside litterbags exposed to solar radiation during the five-month photodecomposition experiment. Data were recorded on one day per month, at three-hour intervals from 7 am to 7 pm, by placing a probe inside each litterbag. Temperature was measured in 15 randomly chosen bags per treatment. The left panel shows data collected in bags from the control (UV exposed) treatment and the right panel shows data from the UV-filtered treatment. There were marked fluctuations in temperatures during the day ($F_{1,1}$ = 10.1, P < 0.01), but there were no significant differences among months ($F_{1,4}$ = 0.53, P = 0.99) or UV treatments ($F_{1,4}$ = 0.11, P = 0.73). Different symbols represent each month (see labels).
