# Supplementary material for: Understanding litter decomposition in semiarid ecosystems: linking leaf traits, UV exposure and rainfall variability
Source: Front Plant Sci. 2015 Mar 17;6:140. doi: 10.3389/fpls.2015.00140 (PMC4362295; doi:10.3389/fpls.2015.00140)
Supplement: Supplementary file 2 [file table_1.docx]

**Table S1 Interspecific comparisons of leaf and litter traits between deciduous *Proustia* and evergreen *Porlieria*.** Values are means ± 1 standard error, and Student’s t tests are shown for interspecific comparisons. ****P* < 0.0001.
